# Maternal Choline and Betaine Supplementation Modifies the Placental Response to Hyperglycemia in Mice and Human Trophoblasts

**DOI:** 10.3390/nu10101507

**Published:** 2018-10-15

**Authors:** Khatia Nanobashvili, Chauntelle Jack-Roberts, Rachel Bretter, Naudia Jones, Kathleen Axen, Anjana Saxena, Kali Blain, Xinyin Jiang

**Affiliations:** 1 Department of Health and Nutrition Sciences, Brooklyn College of the City University of New York, Brooklyn, NY 11210, USA; xatia1988@yahoo.com (K.N.); chauntelle.r@gmail.com (C.J.-R.); bretterrachel@gmail.com (R.B.); jonesnvj@gmail.com (N.J.); kaxen@brooklyn.cuny.edu (K.A.); 2 Department of Biology, Brooklyn College of the City University of New York, Brooklyn, NY 11210, USA; asaxena@brooklyn.cuny.edu; 3 Packer Collegiate Institute, Brooklyn, NY 11201, USA; kblain1@swarthmore.edu

**Keywords:** choline, betaine, gestational diabetes, placental morphology, nutrient transport, vasculature

## Abstract

Gestational diabetes mellitus (GDM) is characterized by excessive placental fat and glucose transport, resulting in fetal overgrowth. Earlier we demonstrated that maternal choline supplementation normalizes fetal growth in GDM mice at mid-gestation. In this study, we further assess how choline and its oxidation product betaine influence determinants of placental nutrient transport in GDM mice and human trophoblasts. C57BL/6J mice were fed a high-fat (HF) diet 4 weeks prior to and during pregnancy to induce GDM or fed a control normal fat (NF) diet. The HF mice also received 25 mM choline, 85 mM betaine, or control drinking water. We observed that GDM mice had an expanded placental junctional zone with an increased area of glycogen cells, while the thickness of the placental labyrinth zone was decreased at E17.5 compared to NF control mice (*p* < 0.05). Choline and betaine supplementation alleviated these morphological changes in GDM placentas. In parallel, both choline and betaine supplementation significantly reduced glucose accretion (*p* < 0.05) in in vitro assays where the human choriocarcinoma BeWo cells were cultured in high (35.5 mM) or normal (5.5 mM) glucose conditions. Expression of angiogenic genes was minimally altered by choline or betaine supplementation in either model. In conclusion, both choline and betaine modified some but not all determinants of placental transport in response to hyperglycemia in mouse and in vitro human cell line models.

## 1. Introduction

Gestational diabetes mellitus (GDM) is a disease characterized by high blood glucose and reduced glucose tolerance that occurs during pregnancy in previously euglycemic women. The incidence of GDM is on the rise in recent years largely due to the epidemic of obesity [1,2]. Gestational diabetes mellitus has negative impacts on both the mother and the infant: GDM-affected women have increased risk of developing type 2 diabetes later in life; GDM also leads to fetal overgrowth or macrosomia (i.e., a birth weight greater than 4 kg) in the neonates, increasing their risks for childhood obesity and metabolic diseases in the future [3,4,5].

The placenta is the organ that mediates nutrient exchanges between the mother and the fetus. Gestational diabetes mellitus is associated with elevated placental transport of macronutrients, a major contributor to the excess accretion of macronutrients by the fetus that results in fetal overgrowth [6,7,8]. Placental nutrient transport is contingent on maternal nutritional status, as well as the placental size, morphology, vasculature (fetal-placental blood flow), and nutrient transporters’ number, density, and activity [9]. The mouse placenta is composed of the decidua (maternal side), junctional zone (the endocrine zone that contains glycogen cells), and labyrinth zone (the vascular zone that mediates fetal-maternal exchange), whereas the human placenta contains decidua, terminal villus unit, and the chorionic plate (fetal side) [10]. Despite the differences in placental morphology, maternal hyperglycemia induces placentomegaly in both species [11,12]. The placental junctional zone is enlarged and glycogen storage increases in rodent placentas, while similarly, the glycogen content surrounding the terminal villi in the human placenta is elevated in GDM [13,14]. Both the labyrinth zone of the rodent placenta and the terminal villus unit of the human placenta have rich maternal and fetal blood flow separated by trophoblasts which mediate the exchange of nutrients and metabolites between the two circulating systems. Gestational diabetes mellitus is associated with increased capillary branching and enlarged surface areas of exchange [15], contributing to greater flows of maternal nutrients which can be taken up by trophoblasts and then transported to the fetal circulation. Both human and rodent placentas have also demonstrated altered expression of macronutrient transporters in GDM, such as the upregulation of glucose transporter 1 (*GLUT1*) which mediates glucose transport and fatty acid binding proteins which mediate fatty acid transport [7,16,17,18]. Reducing placental transport of excess macronutrients to the fetus is a critical step for the prevention of macrosomia secondary to GDM. Normalizing the placental transport in GDM requires the restoration of normal placental morphology and reduction in nutrient transporter activity.

Choline is an essential nutrient that has various functions in cellular membrane structure, cellular signaling, epigenetics, and neurotransmission. When choline is oxidized to betaine, its labile methyl groups become available for methylation reactions. Choline and betaine interact with macronutrient and energy metabolism in multiple ways, and thus may mitigate the macronutrient excess observed in GDM-affected maternal and fetal dyads. For instance, 1-palmitoyl-2-oleoyl-sn-glycerol-3-phosphocholine, a choline-containing phosphatidylcholine (PC), serves as a ligand of peroxisome proliferator activated receptor alpha (PPAR-α) which activates fatty acid oxidation [19]. Betaine is a methyl donor that can epigenetically modify lipogenic genes and suppress their expression [20]. Betaine also enhances mitochondrial respiration to increase energy expenditure [21,22]. Our previous studies have demonstrated that high-fat feeding-induced GDM mice had higher fetal weight in mid-gestation and higher total body adiposity in latest gestation, while maternal choline and betaine supplementation in GDM mice mitigates these indices of fetal overgrowth and excess adiposity [23,24,25]. We further provide evidence to support the potential mechanism in which choline and betaine modify fetal growth in GDM mice via alterations in placental functioning, such as downregulating the placental growth promoter insulin-like growth factor 2 (*Igf2*), suppressing the mechanistic target of rapamycin (mTOR) signaling or reducing macronutrient transporter expression [23,24,25]. Literature also suggests that maternal choline supplementation alters DNA methylation and angiogenesis in human and rodent placentas and/or trophoblasts [26,27,28,29,30].

In this study we further examine the influence of choline/betaine on the determinants of placental transport in greater details with the focus on placental morphology and angiogenic differences among the betaine/choline supplemented and control GDM mice. We corroborate observations in GDM mice with assays in the BeWo choriocarcinoma cell line to investigate the effect of choline or betaine supplementation on nutrient accumulation and transport in human trophoblasts during hyperglycemia. We hypothesize that choline or betaine supplementation would reverse GDM-induced placental morphological and angiogenic changes and alterations in trophoblast metabolism, e.g., enlargement of placental layers, enhanced angiogenesis and angiogenic gene expression, and elevated intracellular glucose and fat accumulation in trophoblasts.

## 2. Materials and Methods

### 2.1. Animal Diet and Treatment

**Figure 1:**
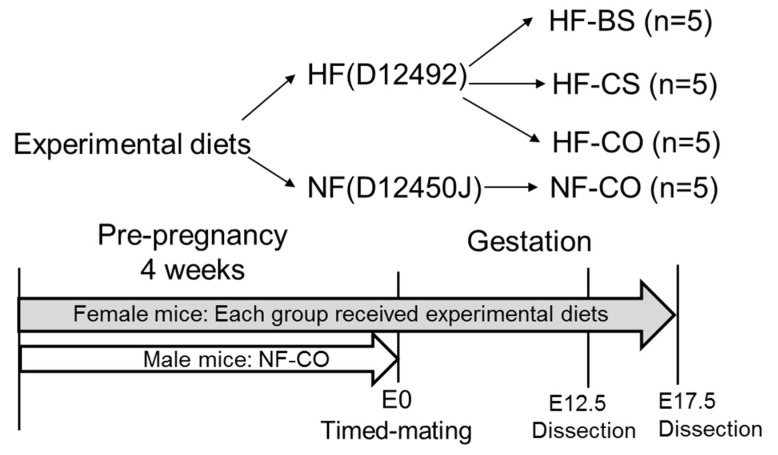
Study design. BS: betaine supplemented; CO: untreated control; CS: choline supplemented; E: embryonic day; HF: high-fat diet; NF: normal-fat diet.

Six-week-old C57BL/6J mice from Jackson Laboratories were housed at conditions of 22 °C, humidity 40–60%, 12 h/12 h light/dark cycle. Female mice (*n* = 5/group) underwent simple randomization and received one of the following diets: normal fat control (NF-CO) which included the D12450J (Research Diets, New Brunswick, NJ, USA) regular mouse diet containing 10% calories from fat and plain drinking water; high fat control (HF-CO) which included the D12492 (Research Diets) high-fat diet with 60% calories from fat and plain drinking water; high fat choline (HF-CS) which included the D12492 HF diet and 25 mM of choline supplemented in drinking water; and high fat betaine (HF-BS) included the D12492 HF diet and 1% betaine (85 mM) supplemented in drinking water. Animals randomized to different groups did not have significant differences in starting weight. Compositions of these diets have been described in a previous publication [24]. The total choline content was 11.7 mmol/kg in the HF diet and 7.6 mmol/kg in the NF diet. Neither diet was deficient in choline. There was no betaine in the diets. The dosages of choline/betaine supplementation were chosen based on prior studies, demonstrating that choline supplementation at the selected dosage improves cognitive development and placental function, while betaine supplementation at the current dosage reduces the hepatic injury due to HF-feeding [29,31,32]. Female mice were fed one of the diets from 4 weeks before timed-mating to embryonic day E12.5 or E17.5 of gestation. Male mice were fed the NF-CO diet for 4 weeks until timed-mating. During timed-mating, two female mice were housed with one male mouse and fed the females’ assigned diet. The presence of a vaginal plug was indicative of successful mating and the date was recorded as E0.5 (Figure 1). If female mice failed to plug after 5 days, timed-mating for the pair would be aborted. We confirmed the impaired glucose tolerance of HF mice via intraperitoneal glucose tolerance tests at E11.5 and E15.5. The glucose tolerance test (GTT) results were published in prior studies [23,24,25].

### 2.2. Sample Collection and Processing

At E12.5 or E17.5, pregnant mice were feed-deprived from 9 a.m. in the morning for 4 h before being euthanized by CO_2_ exsanguination. Each placenta and fetus was dissected from the uterus. Embryo sexing was conducted using PCR of the *Sry* gene on the Y chromosome using a published method [33]. Placentas from one male and one female embryo from each dam were randomly chosen to be fixed in 10% formalin for at least 24 h for histologic analysis. Another two placentas (one male and one female) from each dam were selected randomly and stabilized in RNAlater^®^ (Thermo Scientific, Grand Island, NY, USA) overnight at 4 °C before being stored at −80 °C until gene expression analysis. Only one male pup and one female pup from each litter were randomly chosen for each biological assay because the sample variation inside the litter was much lower than the variation between samples from different litters. The study protocol was approved by the Institutional Animal Care and Use Committee (IACUC) at Brooklyn College (protocol #294).

### 2.3. Placental Histology

Formalin fixed placental samples were sectioned at 5 µm thickness, stained with hematoxylin and eosin, and scanned with the ImageScope software (Leica Biosystems Inc., Buffalo Grove, IL, USA) by Histowicz, Inc. (Brooklyn, NY, USA). Placental slides (2 per dam) from 5 dams of each group were analyzed using the ImageJ software (National Institutes of Health, Bethesda, MD, USA). To measure the thickness of placental layers, the thickest point of the placenta was first identified. The thickness of each layer was then measured and the ratio of thickness of each layer/total thickness of the placenta was measured [34]. To assess the abundance of glycogen cells in the junctional zone, we scanned from one side of the placenta to the other side to capture 10 view field snapshots at 20× magnification that did not overlap with each other. We took equal numbers of snapshots close to the margins of both sides or the center of the placenta in each sample. Thus, the 10 view fields were true representation of the entire tissue section. We measured the area occupied by glycogen cell clusters and divided it by the total area of the junctional zone in the view field. The average ratio from all view fields was calculated for each sample [35]. To assess the labyrinth vasculature, we measured the area of total blood space (including both fetal and maternal sinuses) in the labyrinth zone using the same view field capturing method as what was used for the glycogen cells. The area of blood space was automatically selected by ImageJ using set parameters and adjusted for the total labyrinth zone area in the view field (total blood space/total labyrinth area). The average blood space area was calculated from all view fields for the same sample [36].

### 2.4. Cell Culture and Treatments

The human choriocarcinoma cell line BeWo was retrieved from the American Type Culture Collection (ATCC). Cells were maintained in Kaighn’s Modification of Ham’s F-12 Medium (F-12K Medium, ATCC® 30-2004™, Manassas, VA, USA) and 10% fetal bovine serum (FBS, Mediatech Inc., Manassas, VA, USA) and incubated in 5% CO_2_ + 95% air at 37 °C. During experiments, BeWo cells were cultured in Minimum Essential Medium (MEM, Mediatech Inc., Manassas, VA, USA) containing 2.5% FBS and different glucose and choline or betaine concentrations. The MEM contained 7 μM of choline as was specified by the manufacturer (Corning, Manassas, VA, USA) and the 2.5% FBS contained 16 μM of choline as previously measured [26]. Therefore, the total choline concentration in the MEM and 2.5% FBS was 23 μM. This basal concentration of choline is required to maintain the normal growth and proliferation of trophoblasts without the sign of increased apoptosis observed in trophoblasts with moderate choline insufficiency [26]. The original MEM medium contains 5.5 mM glucose. The glucose concentration of the medium was increased to 35.5 mM to generate high glucose treatment groups. In order to account for the potential change in osmolality due to additional glucose, 30 mM of mannose was added as an osmotic control to the low glucose treatment groups. In summary, there were 4 cell culture conditions during experiments: the normal glucose control group (NG-CO) containing no additional glucose or choline or betaine; the high glucose control group (HG-CO) containing 30 mM additional glucose but no choline or betaine supplement; the high glucose choline supplemented group (HG-CS) containing 30 mM additional glucose and 1 mM choline chloride added to cell culture medium; and the high glucose betaine supplemented group (HG-BS) containing 30 mM additional glucose and 1 mM betaine anhydrous added to cell culture medium. These supplementation levels were selected based on our previous study [37] to maximize the effect of choline or betaine supplementation on endpoints of interest. Cells were seeded at a starting number of 2 × 10^5^ cells per well in 6-well plates in the maintenance medium for 24 h. Thereafter, cells were cultured with one of the four experimental media for 48 h before harvest for RNA extraction. For nutrient uptake experiments, cells were seeded in 96-well plates at a starting amount of 5 × 10^4^ cells in the maintenance medium for 24 h before being switched to experimental media for 48 h. Each experiment was done in triplicate and repeated three times. 

### 2.5. Accumulation of Glucose and Fatty Acid in BeWo Cells

We measured glucose and fatty acid that were taken up by, and accumulated in, the BeWo cells using the commercially available end-point measurement assay kits. The experiments were conducted on cells cultured in 96-well plates after the 48 h treatments with varied levels of glucose and choline or betaine. For the glucose accumulation measurement, cells were transferred to glucose-free MEM containing betaine (1 mM), choline (1 mM), or saline control for starvation and incubated for another 6 h. Thereafter, labeled glucose was measured with the Glucose Uptake-GloTM Assay (Promega, Madison, WI, USA) according to manufacturer’s instructions. This assay uses deoxyglucose (2DG) in place of regular glucose. After the phosphorylation 2DG to 2DG-6-phosphate (2DG6P), G6P dehydrogenase oxidizes 2DG6P and converts nicotinamide adenine dinucleotide phosphate (NADP^+^) to the reduced form of NADP^+^ (NADPH). NADPH provides materials to convert proluciferin to luciferin to be detected using luminescence detection. Luminescence was measured by a luminometer. For the fatty acid accumulation measurement, cells were transferred to glucose-free medium (containing 1 mM betaine, 1 mM choline or saline control) and starved for 4 h. Thereafter, cellular concentrations of the labeled fatty acid were measured after one hour of incubation using the fatty acid uptake kit (Abcam, Cambridge, MA, USA) following manufacturer’s instructions. Labeled dodecanoic acid (C12:0) was used in this assay. Fluorescence was measured at Excitation/Emission = 485/515 nm.

### 2.6. RNA Extraction and Real-Time PCR

RNA was extracted from the RNAlater® stabilized placental samples and freshly harvested BeWo cells using TRIzol reagent (Fisher Scientific, Hampton, NH, USA), reverse transcribed using a High-Capacity cDNA Reverse Transcription kit (Fisher Scientific, Hampton, NH, USA), and analyzed with real-time quantitative PCR as previously described [24]. Expression of the following genes related to placental proliferation (proliferating cell nuclear antigen (*PCNA*)), apoptosis (caspase 3(*CASP3*)), and angiogenesis (vascular endothelial growth factor A (*VEGFA*), placental growth factor (*PGF*), and soluble fms-like tyrosin kinase 1 (*sFLT1*)) were measured in both mouse placentas and BeWo cells. In addition, genes related to glucose and fatty acid transport, such as *GLUT1*, *GLUT3*, fatty acid transporter 1 and 4 (*FATP1* and *FATP4*), and sodium-dependent neutral amino acid transporter-2 (*SNAT2*, encoded by *Slc38a2*) were also analyzed in BeWo cells. Beta-actin (*Actb*) and glucuronidase beta (*GUSB*) were used as housekeeping genes for mouse placentas and BeWo cells, respectively. Primers were either published previously or designed with GeneRunner Version 3.01 (http://www.softpedia.com) (Table S1) [24,26,37].

### 2.7. Statistical Analyses

For mouse placentas, the general linear model (GLM) was used to assess the differences among the groups controlling for embryonic sex and the random effect of different litters. Post-hoc analysis was conducted using the Tukey’s Honest Significant Difference (HSD) test. For the cell culture study, analysis of variance (ANOVA) tests followed by post-hoc Tukey’s HSD tests were conducted to assess the differences among the treatment groups. Data not meeting the normality assumption were log-transformed. All analyses were performed using SPSS (release 24, IBM Inc., Armonk, NY, USA). Differences were considered significant at *p* < 0.05. Values are presented as means ± standard errors (SE).

## 3. Results

### 3.1. Choline and Betaine Supplementation Partially Normalized the Altered Placental Morphology of GDM Mice at E17.5

**Figure 2:**
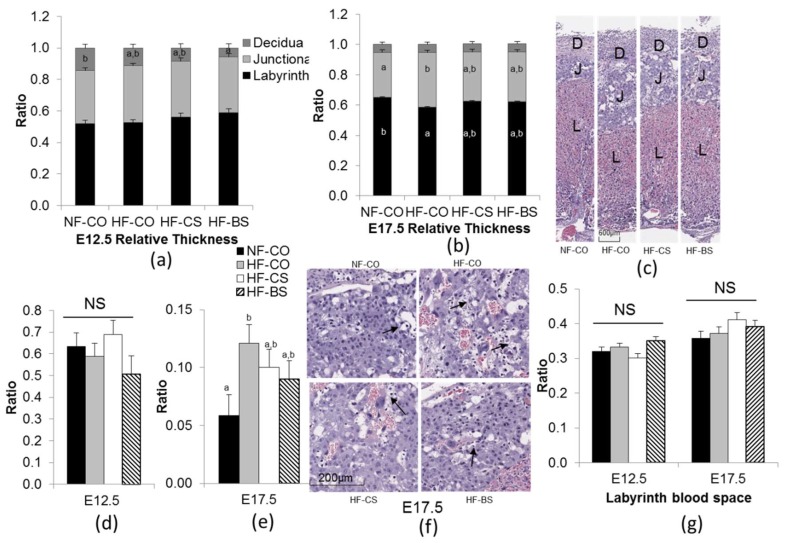
Placental histology at E12.5 and E17.5. (**a**,**b**) Relative thickness of placental layers. (**c**) Histological appearance of representative placentas at E17.5. (**d**,**e**) Glycogen cell area in the junctional zone. Results are presented as glycogen cell area/total junctional zone area. (**f**) Histological images demonstrate representative junctional zones with glycogen cells (arrowheads) at E17.5. (**g**) Labyrinth blood space. Results are presented as labyrinth blood space/total labyrinth layer area. Each group contained 5 dams. Different diets were fed to dams from 4 weeks before timed-mating to gestational day 12.5 or E17.5. Placentas from one male and one female embryo in each dam were included in the analysis. Values are mean ± standard error of mean (SEM); a, b: any two groups with no overlapping characters have a statistically significant difference (*p* < 0.05) between them. D, decidua; J, junctional zone; L, labyrinth zone; NF: normal-fat diet; HF: high-fat diet; BS: betaine supplemented; CO: untreated control; CS: choline supplemented; NS: not significant.

We have previously shown that the HF-fed mice demonstrate impaired glucose tolerance during pregnancy, resembling the characteristics of GDM [23,24,25]. The HF-fed female mice also had higher weight gain and visceral fat weight than the NF-CO mice. Choline or betaine supplementation did not modify the maternal metabolic markers except that choline supplementation improved glucose tolerance at E15.5 [23,24,25]. The choline intake in the HF-CS group (0.66 mmol/week) was approximately 4.5 times of intakes in the CO groups (0.13–0.15 mmol/week). Betaine supplementation led to 1.72 mmol betaine intake per week [23,24,25]. In a previous study, we also observed that maternal HF feeding and choline supplementation did not affect the relative thickness of placental layers at E12.5 [24]. However, in this study, betaine supplementation in the HF-BS group led to lower relative thickness of the decidua than the NF-CO group at this time point (*n* = 5 dams/ group) (Figure 2a). This difference in decidua thickness of the HF-BS versus the NF-CO group was not maintained at E17.5 (Figure 2b,c). The HF-CO group had increased relative thickness of the junctional zone (*p* = 0.02) but decreased relative thickness of the labyrinth zone (*p* = 0.02) versus the NF-CO group (*p* = 0.02) at E17.5 (Figure 2b,c). The HF-CS and HF-BS groups did not show such differences compared to NF-CO, suggesting that the choline or betaine supplementation alleviated the alteration in placental morphology due to HF-feeding. The area of glycogen cell islets in the junctional zone was not different among the groups at E12.5 (Figure 2d), yet was larger in the HF-CO group than in the NF-CO group (*p* = 0.03) at E17.5, while the area in the HF-CS and HF-BS groups did not differ from the NF-CO group (Figure 2e,f). When measuring the area of total blood space in the labyrinth zone as a marker of placental vasculature that determines the surface area of fetal-placental exchange, we observed no differences in this marker among the groups at either E12.5 or E17.5 (Figure 2g).

### 3.2. HF Feeding and Choline/Betaine Supplementation Had Minimal Effects on Placental Angiogenic Gene Expression in GDM Mice

**Figure 3:**
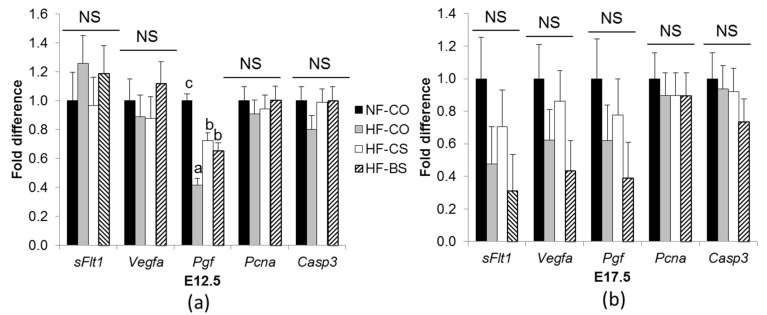
mRNA abundance of angiogenic genes in the placenta at E12.5 (**a**) and E17.5 (**b**). Different diets were fed to dams from 4 weeks before timed-mating to gestational day 12.5 or 17.5. mRNA abundance was measured by real-time PCR. Each group contained 5 dams. Different diets were fed to dams from 4 weeks before timed-mating to gestational day 12.5 or E17.5. Placentas from one male and one female embryo in each dam were included in the analysis. Values are mean ± standard error of mean (SEM); a, b, c: any two groups with different characters have a statistically significant difference (*p* < 0.05) between them. Solid bars: NF-CO; shaded bars: HF-CO; open bars: HF-CS; hatched bars: HF-BS. *Casp3*: caspase 3; *Pcna*: proliferating cell nuclear antigen; *Pgf*: placental growth factor; *sFLT1*: soluble fms-like tyrosine kinase 1; *Vegfa*: vascular growth factor A. NF: normal-fat diet; HF: high-fat diet; BS: betaine supplemented; CO: untreated control; CS: choline supplemented; NS: not significant.

To elucidate the influence of choline/betaine supplementation on placental growth and angiogenesis, we examined the mRNA expression of pro-angiogenic and anti-angiogenic factors as well as proliferative and apoptotic markers. At E12.5, mRNA expression of *Pgf* a pro-angiogenic factor, was downregulated in the HF groups compared to the NF-CO group (*p* < 0.01). Choline and betaine supplementation partially rescued the decrease compared to HF-CO (*p* < 0.05) (Figure 3a). The anti-angiogenic factor *sFlt1*, pro-angiogenic factor *Vegfa* proliferative marker *Pcna* and the apoptotic enzyme *Casp3* had similar expression among the groups. At E17.5, no differences in any of the genes were observed among the groups (Figure 3b).

### 3.3. Choline/Betaine Supplementation Influenced Gene Expression in BeWo Cells under a Hyperglycemic Condition

**Figure 4:**
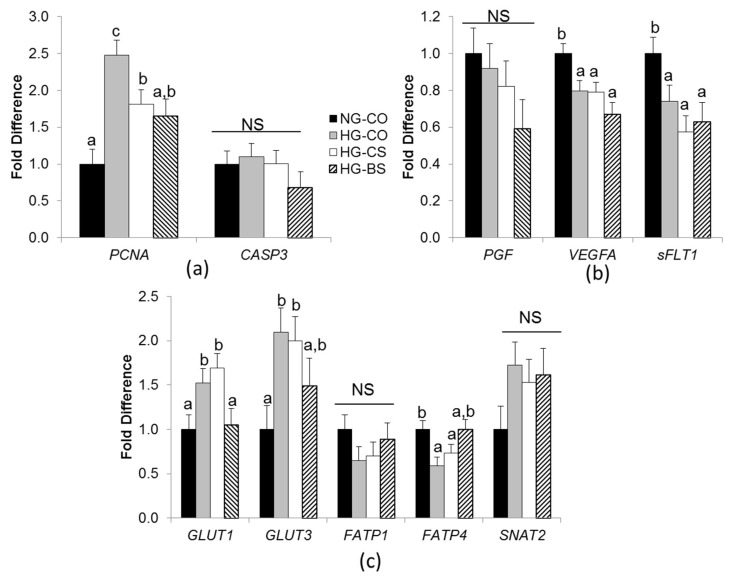
mRNA abundance in BeWo cells. (**a**) Proliferative and apoptotic genes; (**b**) Angiogenic and anti-angiogenic genes; (**c**) macronutrient transporters. BeWo cells were treated with 1mM choline (CS), 1 mM betaine (BS), or saline control and with 30 mM glucose (HG) or 30 mM mannose (NG) in the Minimum Essential Medium for 48 h. mRNA expression was analyzed with real-time PCR. Each experiment was done in triplicate and repeated 3 times. Values are mean ± standard error of mean (SEM); a, b, c: any two groups with no overlapping characters have a statistically significant difference (*p* < 0.05) between them. Solid bars: NG-CO; shaded bars: HG-CO; open bars: HG-CS; hatched bars: HG-BS. *CASP3*: caspase 3; *FATP1*: fatty acid transporter 1; *FATP4*: fatty acid transporter 4; *GLUT1*: glucose transporter 1; *GLUT3*: glucose transporter 3; *PCNA*: proliferating cell nuclear antigen; *PGF*: placental growth factor; *sFLT1*: soluble fms-like tyrosine kinase 1; *SNAT2*: Sodium-dependent neutral amino acid transporter-2; *VEGFA*: vascular growth factor A; NG: normal glucose; HG: high glucose; BS: betaine supplemented; CO: untreated control; CS: choline supplemented; NS: not significant.

Next we examined whether choline/betaine availability ameliorated the influence of hyperglycemia in human trophoblasts using an in vitro BeWo cellular model. Experiments were conducted in triplicate and independently repeated 3 times. High glucose exposure (HG-CO) increased the expression of the cellular proliferation marker *PCNA* (*p* = 0.003) compared to normal glucose control (NG-CO). Choline and betaine supplementation in the HG-CS and HG-BS groups diminished such increases by 30% and 37%, respectively (Figure 4a). However, the cellular apoptotic marker *CASP3* expression was not altered by glucose levels or choline/betaine supplementation. 

We then assessed whether factors that control placental angiogenesis were affected by glucose levels or choline/betaine treatments (Figure 4b). The pro-angiogenic *VEGFA* was downregulated in all HG groups than the NG-CO group (*p* = 0.013), while another pro-angiogenic factor *PGF* was not affected by these treatments. Interestingly, *sFLT1*, an anti-angiogenic factor, also had lower expression in the HG groups than the NG-CO group (*p* = 0.03). Choline or betaine supplementation did not alter the expression of these genes. 

Our previous mouse studies demonstrate that both choline and betaine supplementation can reduce the placental expression of glucose and fatty acid transporters in GDM mice [24,25]. In order to examine whether choline/betaine can exert similar effects on human trophoblasts, we measured the expression of the macronutrient transporters in BeWo cells. The HG-CO group had higher expression of the glucose transporters *GLUT1* and *GLUT3*, but lower expression of the fatty acid transporter *FATP4* than the NG-CO group (*p* < 0.05) (Figure 4c). Betaine supplementation mitigated these alterations in gene expression while choline supplementation did not show any effects. *FATP1* (another fatty acid transporter) and *SNAT2* (an amino acid transporter) expression did not differ in the experimental groups.

### 3.4. Choline or Betaine Supplementation Lowers Glucose and Fatty Acid Accumulation in BeWo Cells

**Figure 5:**
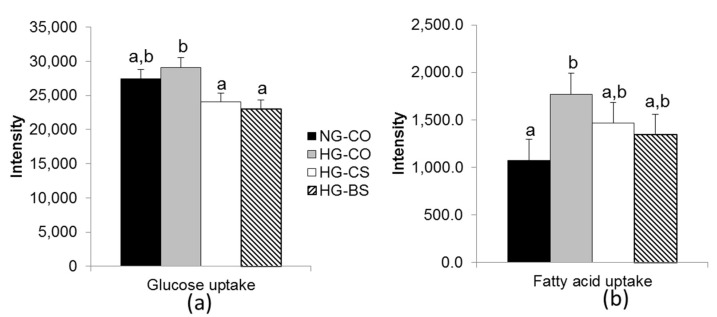
Glucose and fatty acid accumulation by BeWo cells after 1-h incubation. (**a**) The luminescence intensity of labeled glucose that was accumulated in the BeWo cells; (**b**) The fluorescence intensity of labeled fatty acids that were accumulated in the BeWo cells. BeWo cells were treated with 1 mM choline (CS), 1mM betaine (BS), or saline control and with 30 mM glucose (HG) or 30 mM mannose (NG) in the Minimum Essential Medium for 48 h, followed by 1-h incubation with the labeled glucose or fatty acid probes. Each experiment was done in triplicate and repeated 3 times. Values are mean ± standard error of mean (SEM); a, b, c: any two groups with no overlapping characters have a statistically significant difference (*p* < 0.05) between them. Solid bars: NG-CO; shaded bars: HG-CO; open bars: HG-CS; hatched bars: HG-BS. NG: normal glucose; HG: high glucose; BS: betaine supplemented; CO: untreated control; CS: choline supplemented.

To further examine whether macronutrient transport was altered by hyperglycemia and choline/betaine supplementation, we conducted endpoint determination of glucose and fatty acid accumulation in BeWo cells among the different groups after 1 h of incubation with glucose or fatty acid. Both the HG-CS and HG-BS groups had lower intracellular glucose accumulation compared to the HG-CO group (*p* < 0.05) (Figure 5a). Moreover, fatty acid accumulation in BeWo cells over the 1-h incubation was higher in the HG-CO group compared to the NG-CO group (*p* < 0.05), while the accumulation in the HG-CS or HG-BS group did not differ from the NG-CO group, suggesting that intracellular fatty acid accumulation was alleviated by choline/betaine supplementation (Figure 5b).

## 4. Discussion

The current study suggests that hyperglycemia affects placental morphology in late gestation in mice, whereas concurrent choline or betaine supplementation moderately alleviates the morphological alterations. In cultured human trophoblasts, the high glucose treatment alters markers of cellular proliferation, angiogenesis, and nutrient transport. Betaine supplementation seems to have a greater effect than choline on normalizing macronutrient transporter expression, yet both betaine and choline effectively lower macronutrient uptake into trophoblasts.

### 4.1. Choline and Betaine Supplementation Mitigates the Alteration in Placental Layer Thickness in GDM Mice

GDM is a condition associated with excess placental transport of macronutrients to the fetus that results in fetal overgrowth. Placental transport is determined by its morphology, vasculature, and transporter expression and activity. Our mouse study demonstrated that placental morphology was altered by GDM but only in late gestation (E17.5). Hyperglycemia enlarged the relative thickness of the junctional zone and increased the area of glycogen cells in the layer while decreasing the thickness of the labyrinth zone in these mice. The expansion of the junctional zone in diabetic rodents has been reported by other studies and is associated with a larger number of glycogen cells in the layer than euglycemic animals [35]. The number of glycogen cells in the junctional zone normally increases during early and mid-gestation but gradually decreases towards late gestation [35]. However, glycogen cell numbers remain high in GDM mouse placentas in late gestation. The role of the glycogen cells and glycogen accumulation in the placenta is not entirely known. One hypothesis is that placental glycogen storage serves as a reservoir of glucose to be used when fetal-placental demand exceeds maternal glucose supply such as during late gestation [13]. Alternatively, placental glycogen production may serve as a buffering system that prevents excess glucose from being transported to the fetus and accumulated in fetal tissue [38]. The source of glucose for placental glycogen synthesis is also debatable. In human placentas, glycogen is mainly stored around the fetal-placental circulation suggesting glycogen storage as a result of fetal spillover or reverse transport of glucose [38,39]. However, placental glycogen accumulation is also associated with the extent of maternal hyperglycemia, indicating the maternal contribution to placental glycogen storage [14].

This study shows that maternal choline and betaine supplementation attenuated the morphological abnormality of the placental layers and glycogen cell areas arising from GDM at E17.5. We previously observed that both choline and betaine supplementation reduced overall adiposity and hepatic fat accumulation in fetuses of GDM mice at the same time point in late gestation [23,25]. The improvements in fetal metabolism in the betaine and choline supplemented groups may thus serve as a potential mechanism to reduce the amount of glucose being reverse transported from the fetal circulation to the placental junctional zone, thereby mitigating the persistent presence of glycogen cells and enlargement of the placental layer in late gestation. Maternal glucose supply may be another source of glucose for placental glycogen synthesis [38]. However, our previous studies only observed improved maternal glucose tolerance in choline supplemented HF dams while betaine supplemented dams did not show improved glycemia, suggesting that the influence of choline and betaine on maternal glucose control cannot fully explain the normalized morphology in the placental junctional zone of GDM mice [23,25].

In addition to the expansion of the junctional zone, GDM placentas have a relatively smaller labyrinth zone. The labyrinth zone is the main location of maternal-fetal exchange, with rich maternal and fetal blood flow separated by trophoblasts. The shrinkage of the labyrinth zone in GDM mice could be a compensatory mechanism to reduce the amount of nutrients being transported to the fetus, yet a side effect is the reduction in oxygen transport. Hypoxia is common in GDM fetuses since they have an increased demand for oxygen for the excess glucose and fat metabolism [15,40]. The lower fetal and maternal exchange in the labyrinth zone may exacerbate the in utero deficiency in oxygen. Choline and betaine supplementation normalized placental labyrinth thickness, which may improve maternal and fetal exchange. Whether choline/betaine modifies the placental morphology directly or indirectly by normalizing fetal metabolism remains to be explored.

### 4.2. Choline and Betaine Have Minimal Effects on the Vasculature of GDM Placentas

Placental blood flow affects the exchange of nutrients. Previous research suggests that GDM increases measures of angiogenesis by increasing branching of villous capillaries and the diameters of blood vessels; eventually these changes enhance placental blood flow and subsequently nutrient transport [41,42]. However, in the current mouse model we did not observe placental vascular alterations in the labyrinth zone of GDM mice, which may reflect a differential response to hyperglycemia in humans versus rodents. Since placental vasculature is influenced by the action of pro-angiogenic (e.g., *VEGFA* and *PGF*) and anti-angiogenic (e.g., *sFLT1*) factors, we measured angiogenic gene expression in both mouse placentas and human BeWo cells. *Pgf* demonstrated lower expression in GDM mice, while both *VEGFA* and *sFLT1* were downregulated in BeWo cells. The decrease in the pro-angiogenic factors in GDM placentas/trophoblasts corroborates observations in human pregnancies [43,44], although there are also studies with contradictory results [45]. *sFLT1* is shown to increase in diabetic pregnancies [45], thus the cause of its downregulation in BeWo cells treated with high glucose is unclear. Hypoxia and oxidative stress in GDM placentas are potential contributors to the inconsistent alterations in angiogenic factors in different stages of pregnancy and under different disease conditions [15,46]. Choline and betaine had minimal effects on the labyrinth vasculature or expression of angiogenic markers in the rodent and in vitro human trophoblast models of GDM, suggesting that these nutrients influence fetal metabolism and placental function through routes other than angiogenesis in these models. Previous research using the HTR8-SVneo human trophoblast cell line suggests that moderate choline deficiency leads to impaired angiogenesis [26]. However, since in this study all of the groups were choline sufficient, the additional choline or betaine did not seem to have an impact on angiogenesis. 

### 4.3. Both Betaine and Choline Reduce Glucose and Fatty Acid Accumulation in Trophoblasts

Trophoblasts are situated between the maternal and fetal circulation in the placenta and take up both maternal and fetal metabolites. Trophoblast proliferation, transport and uptake affect fetal accretion of nutrients [11]. We previously reported that choline and betaine supplementation reduced the expression of macronutrient transporters such as FATP1 and GLUT1 in GDM mice at mid-gestation and speculated that trophoblast uptake and transport of nutrients would decrease accordingly [24,25]. In the in vitro BeWo cell culture model, we observed that choline and betaine supplementation indeed reduced glucose accumulation and alleviated the increase in fatty acid accumulation in BeWo cells treated with high glucose. Results of this study provide further evidence supporting the hypothesis that choline and betaine reduce accretion of macronutrients by the trophoblasts, thereby alleviating excess macronutrient transfer to GDM-affected fetuses. We also observed an increase in glucose transporter expression in response to hyperglycemia, corroborating findings from our previous mouse study [24]. However, only the betaine supplemented HG-BS group but not the choline supplemented HG-CS group was able to reduce the expression of the glucose transporters. Since both the HG-CS and HG-BS trophoblasts had reduced glucose accumulation, how choline mitigates glucose accumulation in the BeWo cells requires an alternative mechanism than the downregulation of these glucose transporters. It is possible that the localization of glucose transporters to cellular membranes was reduced, thereby reducing the amount of functioning transporters available for nutrient uptake. In fact, maternal choline supplementation in mice suppresses mTOR signaling, a known facilitator of membrane localization of *GLUTs* [47], in mouse placentas [24]. The fatty acid transport *FATP1* was not affected by choline/betaine supplementation, whereas *FATP4* was decreased by HG bu t upregulated by BS. These results also suggest that mechanisms other than transporter expression explain the alleviation of cellular fatty acid accumulation by choline/betaine in BeWo cells. Although *FATP4* was differentially expressed among the groups, since this transporter preferentially transports polyunsaturated fatty acids (PUFA) [48] while the fatty acid uptake assay conducted in this study uses labeled dodecanoic acid (C12:0) as a probe, whether the alteration in *FATP4* expression influences PUFA accretion cannot be assessed by the current method. Since literature suggests that PUFA accretion in GDM-affected fetuses was reduced [49], the higher *FATP4* expression due to betaine supplementation could plausibly have a positive influence on the placental transport of PUFA during hyperglycemia. Betaine and choline supplementation also decreased the expression of the proliferative marker *PCNA* in BeWo cells, which may also reduce proliferation of trophoblasts and their ability to take up macronutrients.

The cellular model has several limitations. It cannot fully account for all pathways of choline metabolism and the crosstalk among different organs that occur in vivo. For example, the enzyme betaine-homocysteine *S*-methyltransferase 1 (BHMT1) is mainly expressed in the liver which transfers the choline or betaine-derived methyl groups to the methionine cycle. The choline or betaine-derived methyl groups can then be incorporated to the universal methyl donor *S*-adenosylmethionine (SAM) and transported to the placenta in vivo, thereby influencing methylation in the placenta. However, the BeWo cells cultured in vitro cannot use the supplemented choline or betaine as a source of methyl groups due to the lack of BHMT in these cells. Therefore, the influence of choline or betaine on the methylation reactions in trophoblasts cannot be determined by the current model. Nevertheless, other influence of choline or betaine on trophoblasts, such as their roles in cellular membrane integrity, PPAR-α activation, or mitochondrial function, is still applicable. We used a high dose of supplementation to maximize any potential effects of choline or betaine on the trophoblasts, yet this dosage was not likely to be achieved physiologically. Dose-response experiments in both in vivo and in vitro systems would be desirable to further delineate the relationship between choline/betaine and trophoblast functioning.

## 5. Conclusions

In conclusion, this study provides in vitro and in vivo evidence regarding the effect of choline/betaine supplementation on the modification of placental functioning during hyperglycemia, by altering placental morphology, lowering macronutrient accumulation, and influencing nutrient transport. 

## Supplementary Materials

The following are available online at http://www.mdpi.com/2072-6643/10/10/1507/s1, Table S1: Primers used for real-time PCR.
