# Supplementary material for: Maternal Choline and Betaine Supplementation Modifies the Placental Response to Hyperglycemia in Mice and Human Trophoblasts
Source: Nutrients. 2018 Oct 15;10(10):1507. doi: 10.3390/nu10101507 (PMC6213524; doi:10.3390/nu10101507)
Supplement: Supplementary file 1 [file nutrients-10-01507-s001.pdf]

Supplementary Information

Table S1. Primers used for real-time PCR[1]

	Forward primer	Reverse primer
	BeWo cells	
CASP3	5′ TAAAATACCAGTGGAGGCCG 3′	5′ TCTCTTTTCTCTGCCTCCACAA 3′
FATP1	5′ GATGGCTATGTCAGCGAGAGCG 3′	5′ TGTAGCCCAGCTCATCCATCA 3′
FATP4	5′ CCCCAGGTGGCTGAGTTCTA 3′	5′ CCGGATGGGGTACACGAAGG 3′
GLUT1	5′ GGCTCCGGTATCGTCAACAC 3′	5′ ATGAGTATGGCACAACCCGC 3′
GLUT3	5′ AACTGGACCTCCAACTTCCTAGTC 3′	5′ GCCAAGAAGGTAATGAGGAAGC 3′
GUSB	5′ CTCTTGGTATCACGACTACGGG 3′	5′ CAATCGTTTCTGCTCCATACTC 3′
PCNA	5′ GGCGCTAGTATTTGAAGCACC 3′	5′ TCTCTTTTCTCTGCCTCCACAA 3′
PGF	5′ TCTCTTTTCTCTGCCTCCACAA 3′	5′ GGCTGGCTTCTCTCTTTCTCTCA 3′
sFLT1	5′ AGGGGAAGAAATCCTCCAGAAG 3′	5′ GTGGTACAATCATTCCTTGTGCT 3′
SNAT2	5′TGGCTGTGACCCTGACAGTACC 3′	5′ ACACTGTAATGAGACTATGACGCC 3′
VEGFA	5′ ACACTGTAATGAGACTATGACGCC 3′	5′ TCTCTTTTCTCTGCCTCCACAA 3′
	Mouse tissue	
Actb	5' TAAGGCCAACCGTGAAAAGA 3'	5' TGCCTGTGGTACGACCAGAG 3'
Casp3	5′ AGCTGGACTGTGGCATTGAGAC 3′	5′ TCCAGGAATAGTAACCAGGTGC 3′
Pcna	5′ GGAGAGCTTGGCAATGGGAACA 3′	5′ GGTACCTCAGAGCAAACGTTAG 3′
Pgf	5′ TGCAGATCTTGAAGATTCCCCC 3′	5′ TCCTCCTTTCTGCCTTTGTCGT 3′
sFlt1	5′ GGGAAGACATCCTTCGGAAGA 3′	5′ TCCGAGAGAA AATGGCCTT TT 3′
Vegfa	5′ CCAAAGCCAGCACATAGGAGAG 3′	5′ TTTGACCCTTTCCCTTTCCTCG 3′

[1] *Actb*: beta-actin; *CASP3*: caspase 3; *FATP*: fatty acid transport protein; *GLUT*: glucose transporter; *GUSB*: glucuronidase beta; *PCNA*: proliferating cell nuclear antigen; *PGF*: placental growth factor; *sFLT1*: soluble fms-like tyrosine kinase 1; *SNAT2*: sodium-dependent neutral amino acid transporter-2; *VEGF*: vascular endothelial growth factor
